# THE STUDY OF KNOWLEDGE AND ATTITUDE ABOUT LONG-TERM CARE AMONG HISPANICS

**DOI:** 10.1093/geroni/igz038.2952

**Published:** 2019-11-08

**Authors:** Ganesh Baniya, Ami Moore

**Affiliations:** University of North Texas, Denton, Texas, United States

## Abstract

Attitudes towards long-term care can help policymakers tailor policies considering different racial and ethnic experiences of the elderly population. The theory of Proactive Coping can help elderly people to better prepare for aging and minimize stressors related to aging by identifying potential sources of stress before they occur and help gather resources and skills for successful aging. The data from the “Long-term care in America: views on who should bear the responsibilities and costs of care” (2017) study showed that Hispanics generally perceived their health to be in a better status and Hispanic women perceived that they had better health compared to males. Similarly, Hispanic males generally thought that they would not need assistance at old age whereas women anticipated that they would require assistance at old age. Similarly, there was a gender difference on who should bear the caregiving responsibility. More Hispanic women thought it would be their responsibility to provide care than males. Women were more prepared than males to provide the care needed to family members of friends. In regards to financial preparation, males reported being more financially capable than females to bear expenses during the old age. Similarly, women were more likely to solely depend on using governmental assistance such as Medicare and Medicaid during old age for needed care. Most of the respondents thought that the US was not well prepared to meet the needs of the aging population and suggested that the government needs to do more before it would be too late.

